# 

**Published:** 1843-05-13

**Authors:** 


☞TO SUBSCRIBERS.—In answer to numerous inquiries from those of our subscribers whose previous punctuality has induced us to deviate partially from our cash principle, we say :—that remittances should be made in *funds current in the State where they reside*, under a postmaster's frank, or in letters *pre-paid*. We would respectfully invite the attention of those subscribers who are in arrears, to the conditions under which they subscribed.

☞ Published every alternate Saturday, and subscriptions received, by J. C. TURNPENNY, N. E. corner of Spruce and Tenth streets. Price, Three Dollars a year, invariably in advance. Two copies sent to the same address, Five Dollars.

Communications and Letters to be addressed (always pre-paid) to the Editor of the Medical Examiner, Philadelphia.

Postmaster's franks can always be obtained for remittances.

This journal is subject to newspaper postage only.

